# Single stage reconstruction of the right thumbnail with Novosorb® Biodegradable Temporizing Matrix (BTM) following massive glomus tumor resection

**DOI:** 10.1016/j.jpra.2025.11.003

**Published:** 2025-11-06

**Authors:** Eimear Phoenix, David Carolan, Robin Caird, Safwat Ibrahim

**Affiliations:** a Royal College of Surgeons, St Stephen’s Green, Dublin, Ireland; b Beaumont Hospital, Beaumont Road, Beaumont, Dublin, Ireland

**Keywords:** Glomus tumor, Nailbed reconstruction, Thumb reconstruction, Biodegradable Temporizing Matrix, Dermal matrices

## Abstract

**Background:**

Glomus tumors are rare, benign vascular neoplasms arising from glomus bodies, often presenting with localized pain and functional limitation. The tumors commonly arise in the sub-ungual region of the distal phalanx and can be locally destructive, posing a unique reconstructive challenge.

**Case presentation:**

We report the case of a 47-year-old male, presenting with a glomus tumor of the distal phalanx of the right thumb, involving more than 70 % of the nailbed resulting in severe pain and functional impairment. Imaging confirmed significant bone erosion of the distal phalanx. Surgical excision with germinal matrix ablation was performed, achieving complete resection. A novel single-stage reconstruction of the right thumb with exposed distal phalanx and nailbed loss was undertaken using a dermal fat graft from the right forearm and Biodegradable Temporizing Matrix (BTM). This approach precluded a complex multi-stage reconstruction and enabled immediate defect coverage and restoration of structural integrity.

**Outcome:**

At 4 weeks review, the BTM had fully integrated and was delaminated to reveal a vascularized wound bed. The wound was left to heal by secondary intention and no skin graft was performed. At 6 months post-operatively, functional outcomes were excellent with restoration of full active range of motion without pain or hypersensitivity.

**Conclusion:**

This case highlights the efficacy of single-stage BTM reconstruction following glomus tumor excision involving the distal phalanx and nail bed. The approach offers a promising alternative to conventional multi-stage reconstruction, ensuring optimal recovery and function. Further studies are needed to validate the efficacy of BTM in hand reconstruction.

## Introduction

Glomus tumors are rare, benign vascular neoplasms arising from glomus bodies, often presenting with localized pain, tenderness, and functional limitation.^1^ These tumors commonly arise in the sub-ungual region of the distal phalanx and can invade locally into bone and the nail bed, posing a unique reconstructive challenge for the discerning hand surgeon.^2^ Whilst surgical excision generally results in resolution, a recurrence rate of 12–33 % is reported and usually results from incomplete excision.^3^ Reconstructive following excision of large digital glomus tumors necessitates the use of intricate local flaps or free flaps via super-microsurgery techniques.^4^ We present the case of right thumb distal phalanx and nailbed reconstruction using single stage BTM following the excision of a large glomus tumor of the right thumb, with dermal fat grafting from the right ulnar forearm in a single surgery. A 6 months post-operatively, the nailbed had fully healed with normal range of motion and no hypersensitivity. To our knowledge, this is the first recorded use of BTM in a single-stage reconstruction of a nail bed. We propose this method as a simple and effective alternative method of nailbed reconstruction.

## Case presentation

**Figure 1:**
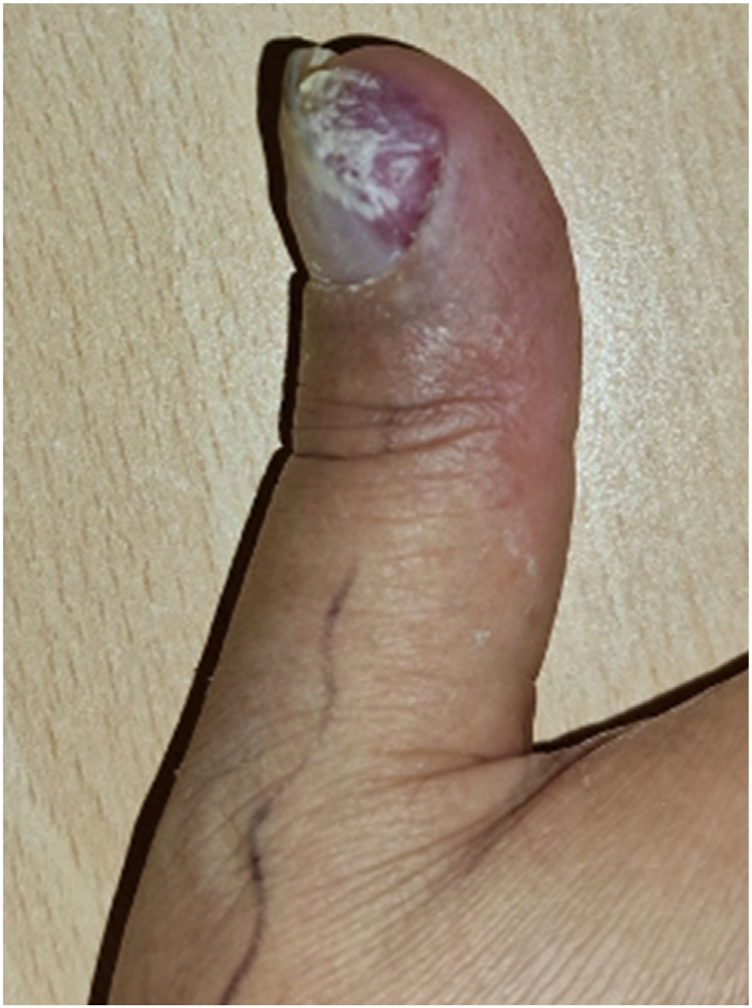
A large glomus tumor of the right thumb causing severe nailbed distortion.

We present the case of a 47 year old right hand dominant man presenting to our plastic surgery clinic with a large subungual tumor involving more than 70 % of the nailbed of the right thumb with significant bony invasion of the distal phalanx (Figure 1). The patient reported the tumor to be present for 12 years, with no history of preceding trauma and having gradually increased in size. The tumor was exquisitely painful over the ulnar aspect of the distal phalanx and was interfering with the patient’s daily activities and quality of life.

Plain film x-ray of the affected thumb demonstrated rounded lucency within the ulnar margin of the terminal tuft and neck of the distal phalanx with overlying soft tissue swelling. A diagnosis of glomus tumor was favored, but an intraosseous epidermal inclusion cyst could not be excluded based on x-ray findings alone. Thus, an MRI with contrast was recommended which confirmed a 20 × 13 × 14 mm lesion suggestive of a glomus tumor. Following discussion with the patient, he was listed for surgical excision of the tumor and reconstruction with BTM.

**Figure 2:**
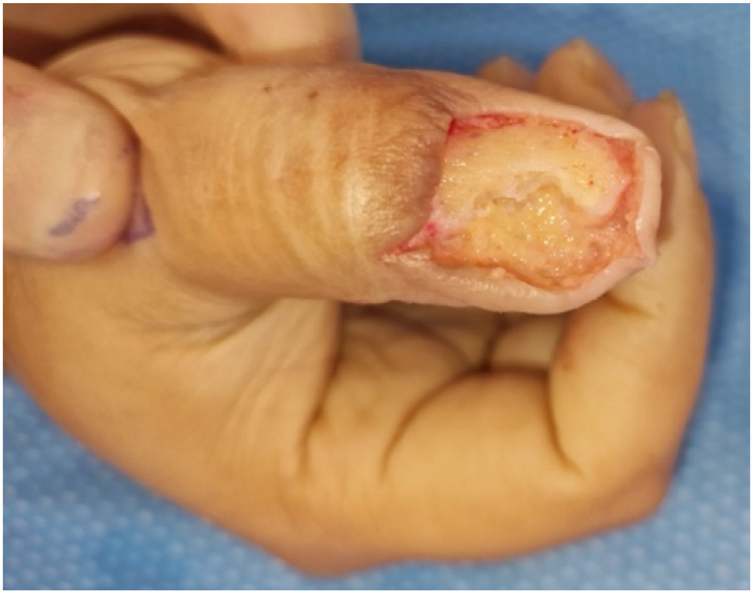
Intra-operative image following glomus tumor excision, burring of the distal phalanx and dermal fat graft placement, prior to BTM inset.

In the operating room, the tumor, sterile matrix and germinal matrix were excised as a single specimen from the right thumb and sent to histopathology with a deep bony margin. It was estimated that the tumor involved more than 80 % of the nailbed, and had eroded through 40 % of the ulnar distal phalanx. The distal phalanx was burred at the site of tumor erosion as well as the dorsal cortex until pinpoint bleeding was established. A dermal fat graft was harvested from the ulnar border of the right forearm and inset into the bony defect of the distal phalanx (Figure 2). BTM was then applied over the dermal fat graft and inset with interrupted 5.0 nylon sutures. The wound was dressed with Polysporin®, Jelonet®, gauze and a zimmer splint secured with cling.

The patient was admitted for 24 h of hand elevation and simple analgesia and was discharged on post operative day 1. Histopathology confirmed the presence of a glomus tumor which was completely excised and without evidence of malignancy.

Two weeks post-operatively, the patient represented with a mild cellulitis of the skin surrounding the surgical site. An explorative surgical washout was undertaken which revealed no pus or collection beneath the eponychial fold or BTM itself. The patient was discharged with a 1 week course of oral co-amoxiclav, the cellulitis successfully resolved and the BTM continued to heal without issue. Although no biopsies were taken to confirm retainment of the lipodermal graft, the patient attended for weekly wound reviews by the operating surgeon and during each inspection the graft was deemed viable. As such, the authors were of the opinion that biopsy confirmation was not required and may cause unnecessary graft disruption as well as posing an infection risk.

**Figure 3:**
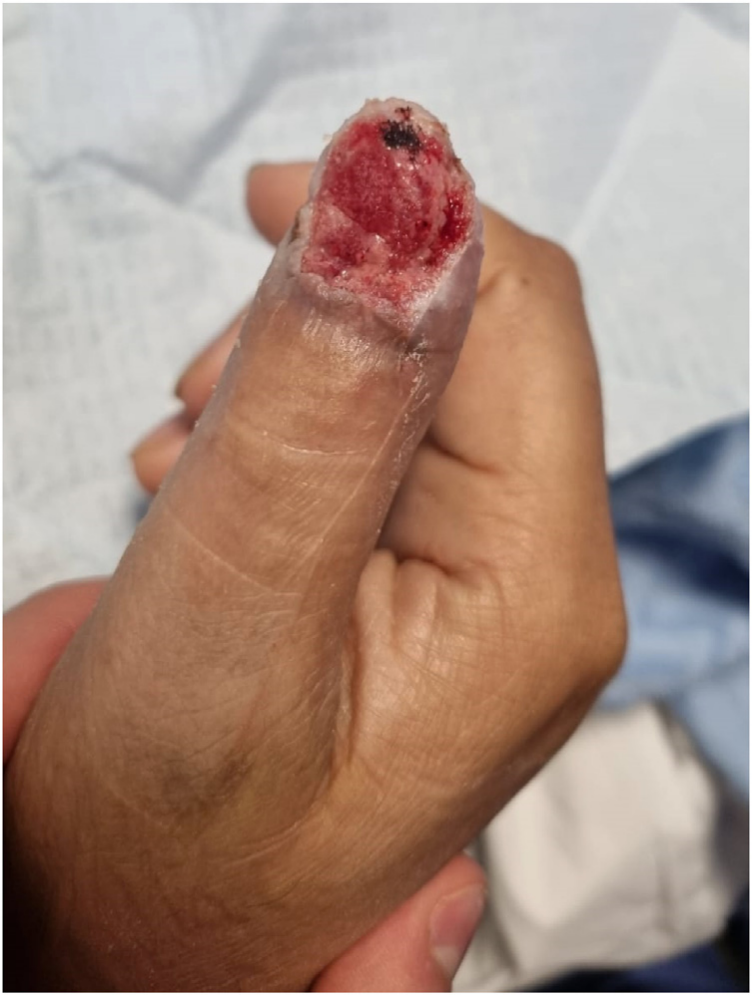
At 4 weeks post operatively the BTM is well integrated and ready for delamination.

**Figure 4:**
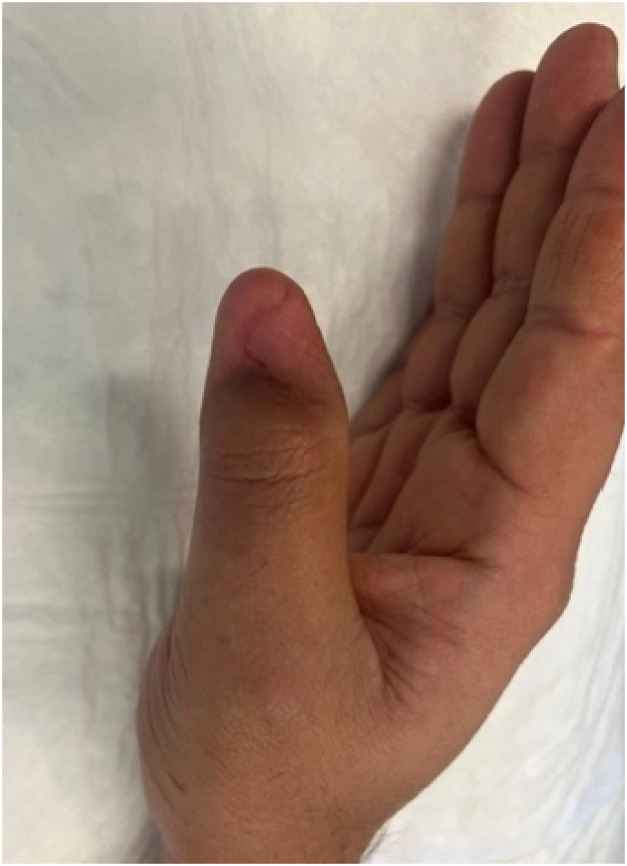
At 6 months following single stage reconstruction of the right thumb using BTM robust soft tissue coverage is achieved.

6 weeks post-operatively, the BTM had fully integrated and was delaminated in the outpatient department to reveal healthy granulation tissue and full bone coverage (Figure 3). At 6 months post-operatively, robust soft tissue coverage of the right thumb without hypersensitivity or pain was achieved (Figure 4). The patient had normal range of motion of the right thumb without restriction of any of his daily activities.

## Discussion

Glomus tumors are rare benign vascular neoplasms which arise from the glomus body, typically located in the sub-ungual region of fingers. Although phenotypically benign, these tumors can cause significant pain and functional limitation.^5^ Surgical excision is the preferred treatment. However, due to the tumor encapsulation, complete resection often results in substantial soft tissue and nail bed defects, as seen in this case. These defects, particularly when involving exposed bone, pose significant challenges for reconstruction. Traditional reconstructive approaches for these tumors typically involve multi-stage procedures involving skin grafts and complex flap reconstructions to achieve adequate coverage while maintaining the functional and aesthetic integrity of the distal phalanx.^3^^,^^4^

The advent of synthetic dermal matrices, such as the Biodegradable Temporizing Matrix (BTM) has presented hand surgeons with a new reconstructive option for complex wounds with large tissue defects. BTM is a synthetic, integrated dermal scaffold designed to promote neo dermal formation by acting as a substrate for cellular infiltration and vascular ingrowth, thereby creating a stable, robust tissue layer over underlying structures.^6^^,^^7^ BTM has the ability to convert previously non graftable wound beds, such as areas of exposed tendon or bone into a surface suitable for skin grafting.^7^ BTM has proven itself to be a reliable and versatile reconstructive option for complex wounds, with a low infection rate, high take rate and excellent graft survival.^8^ Recent studies demonstrate that BTM shows promise in hand reconstruction, particularly in complex cases where graft take is perilous.^9^ Furthermore, it represents a simple, minimally invasive reconstructive option for elderly or comorbid patients who may not tolerate a more robust, uncompromising reconstruction.^7^

While the vast majority of cases reporting the use of BTM involve a two-stage reconstruction, with initial integration of BTM followed by skin grafting or tissue transfer, this case demonstrates its potential future application as a single-stage reconstructive option. We demonstrated how immediate application of BTM following excision of a large glomus tumor in the distal phalanx of the right thumb enabled single-stage reconstruction of the distal phalanx and nail bed by providing a strong and durable scaffold to facilitate wound closure, in an area which previously would have required a complex staged reconstruction.

## Conclusion

To our knowledge this is the first reported case describing single-stage reconstruction of the nail bed using BTM. BTM offers a simple, reproducible and reliable approach to reconstruction, achieving excellent functional and aesthetic results in complex digital reconstructions, ultimately enhancing patient satisfaction and quality of life.

## Funding

None.

## Patient consent

Written consent was obtained from the patient prior to the writing process.

## Declaration of AI and AI-assisted technologies in the writing process

Generative artificial intelligence (AI) and/or AI-assisted technologies were not used in any form in the writing process of this manuscript.

## Conflicts of interests

None.
